# Cathodal transcranial direct-current stimulation over right posterior parietal cortex enhances human temporal discrimination ability

**DOI:** 10.1186/s40101-017-0157-3

**Published:** 2017-12-04

**Authors:** Fuyuki Oyama, Keita Ishibashi, Koichi Iwanaga

**Affiliations:** Department of Design Science, Graduate School of Engineering, Chiba University, 1-33 Yayoicho, Inage, Chiba, Chiba Prefecture 263-8522 Japan

**Keywords:** Time perception, Interval timing, Transcranial direct-current stimulation (tDCS), Posterior parietal cortex (PPC), Duration-discrimination threshold

## Abstract

**Background:**

Time perception associated with durations from 1 s to several minutes involves activity in the right posterior parietal cortex (rPPC). It is unclear whether altering the activity of the rPPC affects an individual’s timing performance. Here, we investigated the human timing performance under the application of transcranial direct-current stimulation (tDCS) that altered the neural activities of the rPPC.

**Methods:**

We measured the participants’ duration-discrimination threshold by administering a behavioral task during the tDCS application. The tDCS conditions consisted of anodal, cathodal, and sham conditions. The electrodes were placed over the P4 position (10-20 system) and on the left supraorbital forehead. On each task trial, the participant observed two visual stimuli and indicated which was longer. The amount of difference between the two stimulus durations was varied repeatedly throughout the trials according to the participant’s responses. The correct answer rate of the trials was calculated for each amount of difference, and the minimum amount with the correct answer rate exceeding 75% was selected as the threshold. The data were analyzed by a linear mixed-effects models procedure.

**Results:**

Nineteen volunteers participated in the experiment. We excluded three participants from the analysis: two who reported extreme sleepiness while performing the task and one who could recognize the sham condition correctly with confidence. Our analysis of the 16 participants’ data showed that the average value of the thresholds observed under the cathodal condition was lower than that of the sham condition. This suggests that inhibition of the rPPC leads to an improvement in temporal discrimination performance, resulting in improved timing performance.

**Conclusions:**

In the present study, we found a new effect that cathodal tDCS over the rPPC enhances temporal discrimination performance. In terms of the existence of anodal/cathodal tDCS effects on human timing performance, the results were consistent with a previous study that investigated temporal reproduction performance during tDCS application. However, the results of the current study further indicated that cathodal tDCS over the rPPC increases accuracy of observed time duration rather than inducing an overestimation as a previous study reported.

## Background

Time perception is indispensable in our daily life. It is a brain function that is necessary not only when synchronizing the timing and rhythm of action but also in many other daily activities, such as controlling the human body to move correctly [1], perceiving speech and utterances [2], and recognizing music and dancing [3, 4]. Because of the importance of time perception, many studies have been conducted to elucidate the neural mechanisms of time perception [5]. However, it still remains unclear how the human brain perceives the flow of time, and the detailed neural mechanisms underlying the human time perception are not yet known. Further investigations are necessary to clarify these mechanisms.

The parietal brain region is generally considered to be associated with many brain functions such as spatial representation, attention and memory [6–8], and the ability to direct attention and attentional distractibility [9–12]. Some have speculated that there is a close relationship between time perception and other brain functions related to the rPPC [13]. Therefore, if such brain function activities change due to certain factors, this may affect not only the performance of the brain function itself but also performance related to time perception (timing performance).

Several studies have shown that the right posterior parietal cortex (rPPC) is one of the areas responsible for the perception of the temporal duration of interval timing, which is a duration range of approximately 1 s to a few minutes. Those studies include brain imaging studies [8, 14, 15], neuropsychological studies [16, 17], and a primate animal study [18] (for reviews see [5, 7, 19, 20]). For example, Oliveri et al. (2009) showed that inhibition of the rPPC caused by repetitive transcranial magnetic stimulation (rTMS) induced an underestimation of time intervals when healthy participants tried to reproduce half-values of standard time durations that were approximately 2 s and were presented by a visual stimulus [17]. In addition to the results of healthy participants whose rPPCs were inhibited by rTMS, Oliveri et al. found that a right brain-damaged patient who had spatial neglect syndrome also underestimated temporal durations in a manner similar to the way that the healthy participants did. Moreover, a right brain-damaged patient without spatial neglect did not underestimate the temporal durations. Notably, the rTMS over the rPPC also distorted the spatial representation of the healthy participants, while it induced an underestimation of time intervals. These results support the hypothesis that the rPPC underlies the processing of the temporal durations of interval timing as well as the processing of spatial information.

Many behavioral and cognitive investigations have used transcranial direct-current stimulation (tDCS) to explore whether changes in brain activities affects behavioral and cognitive performance. With the use of a weak constant electric current, tDCS provides noninvasive brain stimulation that enhances or inhibits the neural activities in a particular brain area of interest [21, 22]. Several studies have found that the direction of the current affects the manner of neural activity alteration; anodal current stimulation is thought to enhance neural activities in the target cortical area, whereas cathodal current stimulation causes an inhibition of neural activities [22, 23]. The effect of current direction is associated with the alteration of neural firing frequency, as anodal current increases neural firing frequency, while cathodal current decreases it [24]. The alteration in the neural firing frequency is considered to be caused by the effect of electric current on the membrane polarization of neurons; anodal tDCS causes depolarization and results in an increased neural firing frequency, while cathodal tDCS hyperpolarizes cortical neurons and decreases the frequency of neural firing [22, 25]. This property of tDCS is useful for testing whether the activity level of a particular brain area affects a certain aspect of behavioral and cognitive performance (e.g., timing performance, attention, memory, and motor control).

If it is found that the change in neural activity affects the performance of a behavioral task, it can be speculated that the stimulated brain region has a relationship with the brain function necessary for performing the task. It had been proposed that anodal tDCS enhances cognitive performance because it causes an excitation of neural activities, whereas cathodal tDCS degrades cognitive performance because it inhibits neural activities. However, this concept has been challenged by several studies [26–28]. For example, Weiss and Lavidor (2012) showed that cathodal tDCS over the rPPC enhanced attentional resources, indicating that cathodal tDCS would not always degrade cognitive performance [26].

Human time perception research has begun to use tDCS to investigate whether changes in brain activities affect timing performance. Vicario et al. (2013) showed that cathodal tDCS over the rPPC affected participants’ performance of a temporal reproduction task [29]. In that task, the participants reproduced the duration of a given standard visual stimulus (standard duration) by pressing a push button. At the beginning of the task trial, each participant observed a standard visual stimulus randomly chosen from 1500, 1600, 1700, 1800, and 1900 msec stimuli. After the presentation of the standard visual stimulus, the participant subjectively reproduced the standard duration with a push button. The durations reproduced by the participant (reproduced duration) were measured, and their average (mean reproduced duration) and coefficient of variation were subjected to the data analysis. The coefficient of variation represents the variability of reproduction, also called as *precision*, which indicates how large the reproduced duration varies across the trials. Note that the value of precision is irrelevant to the difference between the standard duration and the mean reproduced duration. Vicario et al. reported that the participants overestimated the standard durations when cathodal tDCS was applied over the rPPC. This conclusion was based on data showing that the mean reproduced duration under the cathodal tDCS condition was longer than those of the anodal condition and the sham condition. They did not report that the precision of reproduction was affected by the tDCS over the rPPC in any tDCS condition.

The study of Vicario et al. showed that cathodal tDCS over the rPPC led an overestimation, comparing the cathodal condition against the sham condition; however, it did not show the results of comparison between the mean reproduced duration and the standard duration. This comparison is to evaluate the *accuracy* of reproduction, which indicates how close they are between the reproduced duration and the standard duration. From the viewpoint of the difference from the standard duration, it can be said that the reproduced duration in the cathodal condition was almost the same as the standard duration, whereas that of the anodal condition and the sham condition were quite shorter than the standard duration. Therefore, these results could also be interpreted as indicating that the participants reproduced the duration more accurately when cathodal tDCS was applied. However, it was unknown in that study whether tDCS affects the accuracy of subjectively perceived temporal duration, and for this reason further research is needed to investigate this effect of tDCS.

In the present study, to measure the accuracy of timing performance while tDCS was applied over the rPPC, we used a temporal discrimination task to determine the participants’ duration-discrimination threshold. The duration-discrimination threshold is the minimum difference in temporal duration that can be perceived by an observer. A small duration-discrimination threshold is equivalent to a high temporal discrimination performance and therefore to a high timing performance. In the temporal discrimination task trial, each participant observed two visual stimuli and answered which one was presented for a longer duration. We knew that when the two durations are sufficiently different, a participant will perceive the difference and can thus distinguish the two durations. However, if the difference between the two durations is less than the duration-discrimination threshold, the participant will no longer be able to distinguish between the two durations.

In the temporal discrimination task, the difference between the two durations was changed for each trial, and the participant repeated the trial until the threshold could be estimated from the participant’s responses. It is plausible to regard a lower threshold as a higher ability and higher accuracy of timing performance. If the duration-discrimination threshold is low, the participant can distinguish smaller time duration differences and should thus be able to more accurately reproduce the standard duration when performing a temporal reproduction task.

As long as focusing temporal discrimination performance, a temporal discrimination task has advantages over a temporal reproduction task. When an individual’s timing performance is measured with the use of a temporal reproduction task, the duration provided by the participant can vary greatly because the range of response durations is not limited. In other words, the participant can generate arbitrary durations as the reproduced duration, which can cause significant variations in the measurement of reproduced durations. The temporal discrimination task can reduce the variability of the participant’s response because it only requires the participant to indicate the longer one from two durations. Instead, the participant performs trials repeatedly, and the threshold is calculated from data of the entire task. In addition to the advantage described above, there is another advantage that the temporal discrimination task does not require precise physical motor manipulation. In the temporal reproduction task, participants press a push button by the same length as the standard stimulus duration, and thus, the participants need to precisely manipulate their hand in order to press the push button at accurate timing. This property of the temporal reproduction task may cause a confusion between the effects of tDCS because the tDCS may affect both the observation of the stimulus duration and the precision of physical motor manipulation.

## Methods

### Participants

Nineteen healthy volunteers participated in this study (11 males, 8 females, age 23.6 ± 1.2 SD years). We excluded three participants from the analysis: two who reported extreme sleepiness while performing the task, and one who could recognize the sham condition correctly with confidence. Their data showed extremely high thresholds or large variation between conditions, and thus, we considered they should be excluded from the data analysis. For this reason, the data from the remaining 16 participants (10 males, 6 females, age 23.7 ± 1.3 SD years) were used for the further analyses.

All participants had normal or corrected to normal vision. They were all right-handed (handedness score 90.0 ± 9.1 SD), as confirmed by the Edinburgh Handedness Inventory [30]. We explained the purpose, methods, and safety of the experiment to the participants in advance. All participants gave written informed consent prior to the beginning of the experiment, as approved by the Ethics Committee of the Graduate School of Engineering of Chiba University.

### tDCS settings

We used a battery-driven current stimulator (DC-STIMULATOR PLUS, NeuroConn, Ilmenau, Germany) to generate and control the direct-current stimulation. The current was delivered through a pair of sponge electrodes placed on the surface of the scalp. The sponge electrodes were soaked with weak NaCl solution. It was reported that participants feel more comfortable when the electrode solution has a lower concentration (15 mM) compared to a higher one (140 mM) [31]. We therefore used a 0.09% (15.4 mM) NaCl solution, one-tenth of the concentration of physiological saline solution.

The stimulus electrode (5 × 5 cm^2^) was placed over the P4 position defined by the International 10–20 system. According to a study that investigated the relationships between the positions of the cortical regions and the scalp surface by using structural high-resolution MRI [32], the rPPC is located under the P4 position, and thus, current applied over the P4 position is considered to be able to stimulate the rPPC. This notion is supported by a previous study [33], which simulated the electrical field distribution on the cortical surface during tDCS application with computational modeling.

The return electrode (5 × 7 cm^2^) was mounted on the contralateral (left) supraorbital forehead. Several tDCS studies have used a return electrode placed on the supraorbital forehead on the side contralateral to the stimulus electrode [22, 34–36].

The experiment consisted of three tDCS conditions (anodal, cathodal, and sham). The polarity in the condition name was matched with the polarity of the stimulation electrode. In the anodal and cathodal tDCS conditions, the electric current was applied from 5 min before the start of the task to the completion of the task. The intensity of the electric current was 2 mA. In order to reduce the sensation from the current, we gradually ramped up the current for 5 s at the start of the current application and ramped it down at the end of the current application. The current stimulus applied before the start of the task had sufficient duration and intensity to cause its effect by the start of the task [29, 37]. For safety purposes, we set the current stimulator to automatically stop the current 20 min after the start of stimulation. Prior to this experiment, we confirmed that the task was expected to take < 15 min in most cases, and thus, 20 min was adequate for the upper limit of the current duration. The participants were not informed of the current direction throughout the experiment.

In the sham condition, the current was applied in a manner that was identical to the two other conditions but was applied only for the first 40 s and never applied again after being stopped, including the task period. The selection of the real/sham condition was double-blinded.

In light of the tDCS aftereffect (which can last several hours), we had the participants perform each condition on three separate days. In order to reduce the time-of-day effect, which could affect participants’ fatigue and sleepiness, each participant was scheduled to perform each condition at approximately the same time of day (i.e., some participants performed all conditions in the morning, others performed all conditions in the evening). For each participant, the first and the last experimental day were not more than 7 days apart. The order of the three conditions and the polarity of the sham current were counterbalanced across the participants.

### Visual stimulus

The visual stimulus was a green-filled circle (1.8°dia., 20 cd/m^2^) presented by a visual stimulus presentation device that consisted of an LED, light diffuser films, and a light-shielding sheet with a hole of 17.5 mm in diameter. Since the switching of the LED was operated by a microcontroller (Arduino Duemilanove, Arduino), the accuracy of the presentation duration was < 0.1 msec. The participant sat on a stool and put his or her chin on a chin rest, with a viewing distance of 55 cm from the visual stimulus. The lighting in the experimental room was dim (5 lx at viewing position), and a gray curtain was attached to the wall behind the visual stimulus presentation device.

### Task procedure

To estimate the participants’ timing performance, we evaluated the accuracy of the subjective temporal duration (i.e., temporal discrimination performance) by measuring the participants’ duration-discrimination threshold in a temporal discrimination task procedure. Previous studies used this procedure to evaluate their participants’ temporal discrimination performance [38–42].

The task consisted of trials in which the participant compared two durations. At the beginning of the trial, the visual stimulus was presented twice. After this presentation of the visual stimuli, the participant judged which stimulus had a longer duration and indicated it with a push button. If the first stimulus was judged to be longer than the second one, the participant pressed the push button held in his or her left hand, and if the second stimulus was judged to be longer than the first one, the participant pressed the push button held in the right hand. The participant was not informed whether the answer was correct.

The two stimulus durations were 1800 msec and (1800 + *T*) msec, where *T* was a positive variable. They were presented in randomized order, and the interval from the offset of the first stimulus to the onset of the second stimulus was 1.5 s. The duration-discrimination threshold was measured as the minimum *T* value that enabled the participant to perceive the difference between the two stimulus durations. When the participant gave correct responses to > 75% of the trials with a specific *T* value, we considered this *T* value as a perceivable difference. We calculated the correct answer rate of the trials for each *T* value, and we selected the minimum *T* value with the correct answer rate exceeding 75% as the threshold. We determined the thresholds separately for all three tDCS conditions (anodal, cathodal, and sham).

**Fig. 1:**
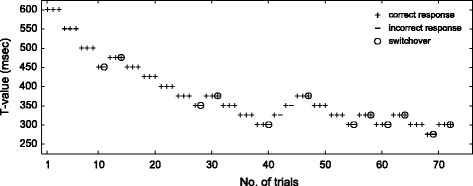
A trace of *T* value in a representative task as an example. This figure shows the *T* value used for each trial in a representative task, performed by one participant in one tDCS condition. A plotting symbol represents whether the response was correct or not: *plus* correct response, *minus* incorrect response. A *circle* indicates a trial in which switching between decrease and increase was observed. In this example, 300 msec was selected as the threshold because it was the minimum *T* value with the correct answer rate exceeding 75% (11 correct responses in 14 trials)

In the task, we repeatedly changed the *T* value depending on the participant’s response in each trial (correct or incorrect). The *T* value was 600 msec in the first trial. Most of the participants easily distinguished the durations with this *T* value. Whenever correct responses were observed consecutively over three trials with the same *T* value, we decreased the *T* value by 25 msec in the next trial, and whenever an incorrect response was observed, we increased the *T* value by 25 msec. Until the first switchover from a decrease to an increase, the *T* value was decreased by 50 msec instead of 25 msec for a quick convergence. The task was terminated when the 12th switch between a decrease and increase was observed. Figure 1 shows a trace of the *T* value in a task performed by one participant in one tDCS condition as an example.

### Data analysis

We assessed the effect of tDCS on the duration-discrimination threshold with the use of a linear mixed-effects models procedure. This procedure is suitable for repeated-measures samples, obtained from within-subject design experiments [43]. The data observed from 16 participants were used in the analysis. The analysis included the tDCS conditions as a fixed effect and the participants as a random effect. We selected the optimal covariance structure model from among first-order autoregressive, compound symmetry, diagonal, and unstructured models. To test whether the covariance structure model is optimal, we used Akaike’s information criterion (AIC) and selected the model with the lowest AIC value. By this procedure, we selected the compound symmetry model as the optimal covariance structure model for the analysis of the duration-discrimination threshold.

We conducted a post hoc multiple comparison test with the Bonferroni adjusted *p* value to estimate the differences in the mean threshold between the tDCS conditions. We also analyzed the differences in the number of trials and the task duration among the tDCS conditions. These analyses also used a linear mixed-effects model procedure. Based on the AIC value, the diagonal model was selected as the optimal covariance structure model for the analyses of the number of trials and the task duration. The statistical analyses were performed with IBM SPSS Statistics (ver. 19) software (IBM Corp., Armonk, NY).

## Results

**Fig. 2:**
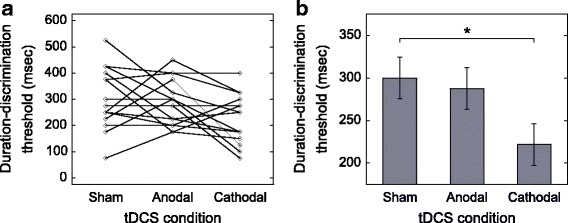
The duration-discrimination threshold observed for each tDCS condition (sham, anode, and cathode). Individual duration-discrimination thresholds for three conditions (**a**); the mean duration-discrimination threshold for each tDCS condition (**b**). The participants performed all of the tDCS conditions in the within-subject experimental design. The statistical analysis detected a significant difference between the sham and cathodal tDCS conditions (*p* = 0.041), indicating that cathodal tDCS enhanced the participants’ duration-discrimination ability. Error bars indicate the standard error of the mean

We analyzed the data obtained from the 16 participants. The mean duration-discrimination threshold value and standard deviation for the three conditions were as follows: sham, 300.0 ± 113.7 msec; anodal tDCS, 287.5 ± 85.6 msec; and cathodal tDCS, 221.9 ± 91.7 msec. We tested the mean duration-discrimination threshold values by the linear mixed-effects model procedure, and the standard error and the degree of freedom were calculated to be 24.434 and 39.841 for each of the tDCS conditions, respectively. Our analysis of the duration-discrimination thresholds indicated a significant effect of the tDCS conditions (*F*(2, 30.000) = 3.957, *p* = 0.030). The post hoc test revealed a significant difference between the sham and cathodal tDCS conditions (*p* = 0.041). No significant differences were detected between the sham and anodal tDCS conditions (*p* = 1.000) or between the anodal and cathodal tDCS conditions (*p* = 0.107). The mean values and their standard errors are illustrated in Fig. 2.

We conducted all tDCS conditions in a counterbalanced order, and thus, each participant underwent one of the three conditions first, and although it was ideal that all three tDCS conditions had the same chance to be performed as the first condition, the number of participants who underwent each condition first differed slightly. Seven participants underwent the sham tDCS condition first, four underwent the anodal condition first, and five underwent the cathodal condition first. However, we did not find any differences in the threshold value between the sham and anodal conditions despite these differences, and thus, we considered that the differences in the first-performed condition did not affect the threshold values.

The average number of trials and standard deviation were as follows: sham, 70.0 ± 12.9; anodal tDCS, 71.5 ± 8.0; and cathodal tDCS, 79.4 ± 13.9. Based on the analysis results, the standard error for the sham, anodal, and cathodal conditions was calculated to be 3.217, 2.000, and 3.480, respectively, and the degree of freedom was 15.000 for all conditions. We analyzed the differences in the trial number among the tDCS conditions by the linear mixed-effects model procedure, and it detected no significant differences (*F*(2, 34.920) = 2.372, *p* = 0.108).

The average duration of the task and standard deviation were as follows: sham, 569 ± 100 s; anodal tDCS, 578 ± 55 s; and cathodal tDCS, 649 ± 121 s. Consequently, since the duration of tDCS application was 5 min longer than the task duration, the mean durations of tDCS application were 14 min 29 s, 14 min 38 s, and 15 min 49 s, respectively. Based on the analysis results, the standard errors for the sham, anodal, and cathodal conditions were calculated as 24.955, 13.806, and 30.287 s, respectively, and the degree of freedom was 15.000 for all conditions. The analysis of the task duration indicated no significant differences (*F*(2, 29.410) = 2.582, *p* = 0.093). The duration of the tDCS application did not exceed 20 min for all participants in all tDCS conditions.

## Discussion

Our present findings revealed that the duration-discrimination threshold was lower in the cathodal condition compared to the sham condition, i.e., the threshold was lower when the cathodal current stimulation was applied over the rPPC. This implies that cathodal tDCS over the rPPC enhances human temporal discrimination performance. In the study by Vicario et al. (2013) that investigated the effects of tDCS on their participants’ temporal reproduction performance, the participants were instructed to reproduce the same duration as the standard duration during tDCS application [29]. Vicario’s research group showed that the cathodal tDCS over the rPPC resulted in a longer reproduced duration compared to the anodal and sham tDCS conditions. However, the reproduced duration in the cathodal tDCS condition was comparable to the standard duration, whereas the reproduced duration in the anodal and sham tDCS conditions appeared shorter than the standard duration. The results could thus also be interpreted as indicating that the reproduction was more accurate under the cathodal tDCS condition. Considering the previous results from this view point, we find them consistent with our present findings, supporting the notion that cathodal tDCS over the rPPC enhances temporal discrimination performance.

Contrary to the previous findings that cathodal tDCS has an inhibitory effect since it decreases the neural firing frequency [22–24], the cathodal tDCS used in our present study appeared to have the effect of enhancing the participants’ temporal discrimination performance. Although it had been assumed that cathodal tDCS degrades cognitive performance because it inhibits neural activities, this has been challenged by several studies. For example, Weiss and Lavidor (2012) showed that cathodal tDCS over the rPPC enhanced attentional resources, indicating that cathodal tDCS would not always degrade cognitive performance [26]. Berryhill et al. (2014) summarized previous studies investigating the effects of tDCS on cognitive performance with a focus on working memory and found that cathodal tDCS could result in improvement of cognitive task performance depending on stimulation site [27]. Moreover, Focke et al. (2017) reported that only cathodal tDCS had the effect of stabilizing previously learned motor sequences [28]. These findings showed that cathodal tDCS has enhancing as well as inhibitory effects on cognitive performance. In addition, the effects of tDCS on brain functions depend not only on the current direction but also on the placement of electrodes, current intensity, and size of electrodes. With a certain placement, intensity, and size, even cathodal tDCS can enhance cognitive performance.

Our present findings are consistent with these studies, which showed that cathodal tDCS does not always impair cognitive performance and can even improve brain function. Our results can thus be interpreted as indicating that cathodal tDCS enhances the excitability of the rPPC and leads to an improvement in timing performance.

However, it is unknown whether inhibition of neural activity always impairs brain function. For this reason, we can also hypothesize that the timing performance was enhanced despite the inhibited neural activity of the rPPC resulting from the decrease in the neural firing frequency caused by the cathodal tDCS. As an explanation of why inhibition of the rPPC resulted in enhanced timing performance, we speculate that an inhibition of neuronal firing in the rPPC decreased attentional distractibility and thus enhanced the temporal discrimination performance. This speculation is based on the previous findings that the parietal brain region is related to the function of directing attention and attentional distractibility. Several studies showed that the rPPC is involved in directing attention to an object in the environment [9–11]. Hayashi et al. (2014) showed that participants who had smaller rPPC gray matter volume performed better at a temporal discrimination task [15], and the authors considered that a large gray matter volume in a particular brain area does not always provide excellent brain function involving neural activity in that area. Hayashi’s research group also showed the possibility that the individual differences in temporal discrimination performance resulted from the difference in attentional distractibility. This possibility was based on a previous finding that a lower attentional distractibility score is associated with a smaller gray matter volume of the parietal region [12].

Since the volume of the gray matter of the parietal region is related to the degree of attentional distractibility, we speculated that the neural activity in the parietal region is related to the function of directing attention to other objects. If this speculation is true, it may be possible that when cathodal tDCS suppresses the neural activity in the parietal region, the attention of the participant is steadily directed to the task and it is difficult to direct attention to other objects. In addition, this notion is supported by an evidence that anodal tDCS over the rPPC improved the participants’ attentional capability and their visuospatial performance to detect visual stimuli located across the visual field [44]. This evidence can be interpreted as indicating that anodal tDCS enhanced the neural activity in the parietal region, directed the attention throughout the visual field, and resulted in the improved visuospatial performance. However, it was also reported that cathodal tDCS over the rPPC enhanced attentional capacity resources rather than simply change attention allocation between center and periphery [26]. Considering these findings together, we might be possible to speculate that cathodal tDCS over the rPPC interferes the changing of attention allocation between mental matters in one’s mind (i.e., what you are concentrating on thinking in your mind) while the balance of attention allocation between visuospatial locations (i.e., center and periphery) was not affected. Based on this speculation, cathodal tDCS prevents attention from directing to mental matters irrelevant to the task, and it resulted in improved task performance. Although these speculations we discussed above can consistently explain the present results, further investigations are necessary to test the validity of these speculations because we did not directly measure the participants’ attentional abilities or attentional distractibility.

We observed the effect of cathodal tDCS on temporal discrimination performance, but we did not observe the effect of anodal tDCS. This is in agreement with the study by Vicario et al. (2013), which showed that cathodal tDCS affects participants’ time reproduction performance, while the effects of anodal tDCS were not detected. Although it remains unclear why no effects of anodal tDCS were observed in the present study, we suggest several hypotheses.

The first is that the size of the effect might depend on the current’s direction, i.e., the cathodal tDCS remarkably decreased the neural firing rate whereas the anodal tDCS did not cause much of an increase. For example, if the cells had already been firing at a relatively high frequency, it is possible that anodal tDCS did not increase the neural firing frequency much. This might be influenced by physical factors such as the relationship between the firing threshold potential and the resting potential and the ionic balance between the inside and the outside of the neuronal cell membrane [45–47].

A second possibility is that the neural firing rate actually increased but did not greatly affect the capability of directing attention. In other words, changes in directing attention capability might be more sensitive to a decrease of neural firing frequency than to an increase. There might be a ceiling effect that makes it impossible for the anodal tDCS to further enhance the capability of directing attention. It is also possible that changes in the threshold were not detectable in the current task due to their slightness even though the anodal tDCS did actually affect the capability of directing attention.

A third possibility is that the anodal tDCS affected the capability of directing attention but the amount of attention increased equally for all objects, including peripheral objects and the task itself. If the anodal tDCS enhanced the capability of directing attention, it could result in an increase in the amount of attention to the periphery, but simultaneously, it might also cause an increase in the amount of attention to the task itself. It can thus be speculated that the effect of the anodal tDCS was not detectable because the relative amount of attention to the periphery was not changed. In relation to this possibility, it was reported that anodal tDCS over the rPPC improved the participants’ attentional capability and their visuospatial performance to detect visual stimuli located across the visual field [44]. This finding supports the possibility that anodal tDCS enhanced peripheral attention, as well as central attention.

Although we suggest some of the possible explanations listed above, the present study did not use a task to measure attentional allocation or distraction. For this reason, further research is needed to confirm the validity of the above-described hypotheses and to address the remaining unclear points regarding the precise mechanism by which tDCS affects human brain function.

In the present study, the number of trials depended on the participant’s responses, so that the task duration was not controlled by the experimenter. Although there were no significant differences in the number of trials or the task duration, the data showed tendencies for differences between the conditions. Apart from statistical significance, the cathodal condition seemed to have more trials and a longer task duration compared to the two other conditions. Considering this together with the other results, the lower threshold tended to be associated with a larger number of trials. The reason for this is the nature of the task, i.e., the *T* value started from a large value and decreased until it reached the threshold. As a consequence of the nature of the task, there was a tendency for more trials to be required to reach a low threshold, which was largely different from the initial *T* value. For this reason, even if there was a tendency for a change in the number of trials, it is still appropriate to conclude that the cathodal tDCS lowered the participants’ duration-discrimination threshold.

The tDCS return electrode was mounted on the contralateral (left) supraorbital forehead in the present study according to the previous studies. This might result in a confounding effect that tDCS could stimulate the location that the return electrode was placed over. However, the area of the return electrode (5 × 7 cm^2^) was larger than that of the stimulus electrode (5 × 5 cm^2^) so that the density of the current that passed through the return electrode was smaller than that of the stimulus electrode. For this reason, the current near the return electrode was less effective than the current passing through the stimulus electrode, and thus, it is more reasonable to conclude that the results in the present study were caused by the change of activity in the rPPC, which the stimulus electrode was placed over.

We cannot positively assert that the current affected only the rPPC and therefore need to consider the relationships between brain-area function and task property, as well as the electrode position. In light of the many previous studies that revealed the relationship between the rPPC and human temporal performance, however, it is plausible to consider that the tDCS affected rPPC and resulted in enhanced temporal discrimination performance.

## Conclusions

In summary, we investigated human time perception from the viewpoint of brain activity by administering tDCS and found a new effect that cathodal tDCS over the rPPC enhanced temporal discrimination performance. In terms of the existence of anodal/cathodal tDCS effects on human timing performance, our results here were consistent with those of a previous study that investigated temporal reproduction performance during tDCS application. However, our results further indicated that cathodal tDCS over the rPPC increased accuracy of observed time duration, rather than inducing an overestimation, as was reported in a previous study. Although we observed that cathodal tDCS enhanced temporal discrimination performance, the mechanisms underlying this effect remain unknown. Confirmation of the validity of the hypotheses we have described herein requires further research including measurements of brain functions other than temporal perception, such as spatial representation, memory capacity, and attentional allocation and distractibility.

## Abbreviations

AIC: Akaike’s information criterion
rPPC: Right posterior parietal cortex
rTMS: Repetitive transcranial magnetic stimulation
tDCS: Transcranial direct-current stimulation
